# Viral oncogenes, viruses, and cancer: a third-generation sequencing perspective on viral integration into the human genome

**DOI:** 10.3389/fonc.2023.1333812

**Published:** 2023-12-21

**Authors:** Ruichen Ye, Angelina Wang, Brady Bu, Pengxiang Luo, Wenjun Deng, Xinyi Zhang, Shanye Yin

**Affiliations:** ^1^Department of Pathology, Albert Einstein College of Medicine, Bronx, NY, United States; ^2^Einstein Pathology Single-cell & Bioinformatics Laboratory, Bronx, NY, United States; ^3^Stony Brook University, Stony Brook, NY, United States; ^4^Tufts Friedman School of Nutrition, Boston, MA, United States; ^5^Horace Mann School, Bronx, NY, United States; ^6^Tongji Medical College, Huazhong University of Science and Technology, Wuhan, China; ^7^Clinical Proteomics Research Center, Massachusetts General Hospital, Harvard Medical School, Boston, MA, United States; ^8^Department of Respiratory Diseases, The Second Affiliated Hospital of Nanchang University, Nanchang, China

**Keywords:** viral integration, cancer sequencing, third generation sequencing, nanopore, PacBio

## Abstract

The link between viruses and cancer has intrigued scientists for decades. Certain viruses have been shown to be vital in the development of various cancers by integrating viral DNA into the host genome and activating viral oncogenes. These viruses include the Human Papillomavirus (HPV), Hepatitis B and C Viruses (HBV and HCV), Epstein-Barr Virus (EBV), and Human T-Cell Leukemia Virus (HTLV-1), which are all linked to the development of a myriad of human cancers. Third-generation sequencing technologies have revolutionized our ability to study viral integration events at unprecedented resolution in recent years. They offer long sequencing capabilities along with the ability to map viral integration sites, assess host gene expression, and track clonal evolution in cancer cells. Recently, researchers have been exploring the application of Oxford Nanopore Technologies (ONT) nanopore sequencing and Pacific BioSciences (PacBio) single-molecule real-time (SMRT) sequencing in cancer research. As viral integration is crucial to the development of cancer via viruses, third-generation sequencing would provide a novel approach to studying the relationship interlinking viral oncogenes, viruses, and cancer. This review article explores the molecular mechanisms underlying viral oncogenesis, the role of viruses in cancer development, and the impact of third-generation sequencing on our understanding of viral integration into the human genome.

## 1 Introduction

Cancer is a complex and multifaceted disease with genetic alterations playing a central role in its initiation and progression. It is comprised of genetically diverse heterogeneous groups of diseases that are constantly evolving, stretching across populations and individuals (1–6). Throughout the years, several studies, such as the PCAWG Project (7), have investigated these variations or mutations in cancer cells to see the relationship between the genetic changes and their role in cancer (1, 8, 9). Although somatic mutations have traditionally been the focus of cancer genetics, viral infections also contribute significantly to the global cancer burden (10–12). 15.4% of all cancers are attributable to infections and 9.9% are linked to viruses, according to the World Health Organization (WHO). After Helicobacter pylori, the four most prominent infection-related causes of cancer are HPV, HBV, hepatitis C, and EBV. These viruses are widely known to the general population and, with their rising infection rates, are to be a significant matter to raise up. Viruses are part of a multistep pathway of oncogenesis and contribute to the development of cancer (8, 12). Characteristics that can be attributed to the development of cancer through human viruses include the presence and persistence of viral DNA in tumors, growth-promoting activity of viral genes in model systems, malignant phenotype dependence on viral oncogene expression, and epidemiological evidence that viral infection can be a major risk of cancer development (11). Recently, there have been efforts made by the worldwide scientific community to specifically analyze comprehensive genomes and whole transcriptomes from tissue samples from cancer patients. The PCAWG Project, for example, collected whole genome sequencing data from 2,658 cancers in 38 tumor types, most likely from third-generation sequencing, a new and uprising technique being used to sequence genomes (8, 11, 13–15). This review aims to provide an overview of viral oncogenes, their role in cancer, and the recent advancements in third-generation sequencing that have enhanced our ability to study viral integration into the human genome.

## 2 Viral oncogenes and their role in cancer

Viral oncogenes are genes carried by certain viruses that can promote cellular transformation and lead to the development of cancer. These genes are often homologous to cellular proto-oncogenes, but their regulation and expression are altered in infected cells. In these infected cells, normal cell growth control mechanisms are disrupted, and they display uncontrolled proliferation. Tissue-specific functions cease and ultimately lead to the development of cancer. 15-20% of all human cancers have been caused via infection by oncogenic viruses (12). Key viral oncogenes include E6 and E7 from human papillomavirus (HPV), E1A and E1B from adenovirus, and Tax from human T-cell leukemia virus (HTLV-1). These oncogenes disrupt normal cellular processes, such as cell cycle regulation, apoptosis, and DNA repair, leading to uncontrolled cell growth and eventual tumor formation.

### 2.1 Viruses implicated in human cancer

Several viruses have been linked to the development of various cancers. Notable examples include:

#### 2.1.1 Human papillomavirus

HPV is a DNA virus associated with cervical, anal, and oropharyngeal cancers. More than half of all malignancies related to infection around the world are caused by HPV, with an incidence of approximately 5% among all cancers worldwide (16–18). HPV16 and HPV18, with less frequency, are involved in a subset of head and neck cancers (HNCs) which are called oropharyngeal squamous cell carcinomas (OPSCCs) (19, 20). The fraction of OPSCCs arising from HPV infection has been around 20%. Integration of HPV DNA into the host genome disrupts tumor suppressor genes and promotes oncogene expression. HPV carries two critical oncogenes: E6 and E7. E6 promotes the degradation of the tumor suppressor protein p53, and E7 disrupts the function of the retinoblastoma protein (pRb), leading to uncontrolled cell division (17, 21, 22). Specifically, hrHPV E6 binds to p53 and promotes its degradation, triggering uncontrolled cell proliferation through cell cycle checkpoint evasion. HPV+ cancer cells have been shown to retain unaltered p53 and pRb genes, which is unlike most human tumors containing mutations in these tumor suppressor genes. Understanding the molecular mechanisms of these oncogenes is crucial for developing targeted therapies against HPV-associated cancers.

#### 2.1.2 Hepatitis B and C viruses

Viral hepatitis stemming from the hepatitis B and C viruses is primarily associated with severe health complications, including liver cirrhosis, hepatocellular carcinoma, hepatic fibrosis, and steatosis. Both are considered to be a major healthcare problem and are blood-borne viruses that primarily infect the liver. Hepatitis B is a life-threatening liver disease caused by the highly contagious viral pathogen called hepatitis B virus (HBV) (23–25), which is an enveloped virus belonging to Hepadnavirida. Hepatitis C virus (HCV) is a positive-stranded RNA virus that causes severe liver disorders and is related to Togaviridae or Flaviviridae. It is a small spherical enveloped virion with an icosahedral capsid (26–28). Chronic hepatitis B and C viral infections can result in hepatocellular carcinoma (HCC) through mechanisms involving viral integration and chronic inflammation. Integration of viral DNA into the host genome can cause genomic instability and chronic inflammation, promoting the progression of liver cancer. Studying these viral integration sites and their impact on host genes is essential for identifying potential therapeutic targets. It is crucial to implement preventative strategies and therapies to eradicate chronic infection of HCV and suppress viral replication for HBV (26, 27).

#### 2.1.3 Epstein-Barr virus

Epstein-Barr Virus, a member of the herpesvirus family, is associated with several cancers including nasopharyngeal carcinoma, Burkitt’s lymphoma, and gastric carcinoma. EBV infects B cells and epithelial cells, maintaining itself in a latent state in B cells for the lifetime of the host (29–32). The association of EBV with various human cancers has been well-documented. The virus encodes several genes that promote cell growth and inhibit apoptosis, such as LMP1 and EBNA2. LMP1 acts as a constitutively active receptor, activating several signaling pathways leading to cell proliferation and survival. EBNA2, on the other hand, controls the expression of genes promoting B cell growth (33–35). While integration of the EBV genome into the host chromosome isn’t common, the expression of its viral oncogenes and their interaction with cellular pathways plays a pivotal role in the development of EBV-associated cancers (36–40).

#### 2.1.4 Human T-cell leukemia virus

HTLV-1 is a retrovirus known to cause adult T-cell leukemia/lymphoma (ATL). This virus integrates its DNA into the host genome, leading to the clonal expansion of infected cells (41). HTLV-1 is a key viral oncogene encoding for Tax protein, which can activate a variety of cellular pathways leading to cell proliferation. While the precise factors contributing to the varied outcomes of HTLV-1 infection remain incompletely elucidated, mounting evidence indicates that a intricate interplay between the virus and the host, along with the host’s immune response to HTLV-1, likely govern the emergence of HTLV-1-associated diseases (42–44). By disrupting cell cycle regulation and DNA repair mechanisms, Tax promotes genetic instability and the survival of damaged cells, paving the way for leukemogenesis. In some cases, HTLV-1-associated adult T cell leukemia/lymphoma can present with unique clinical manifestations, such as granulomatous pneumocystis jiroveci pneumonia and hypercalcemia.

### 2.2 Viral integration into the human genome

The contribution of viruses to carcinogenesis has been a topic of profound significance in the realm of cancer biology. Central to this paradigm is the mechanism through which viruses integrate their DNA into the human genome (45–47). Such integration events play a pivotal role in the life cycle of oncogenic viruses, often precipitating a cascade of cellular disruptions that culminate in tumorigenesis. Timely identification of these human-infecting viruses is crucial for understanding their role in carcinogenesis and for implementing appropriate interventions (48). Recent advancements have enabled rapid identification of such viruses, providing a more comprehensive understanding of their implications in human health.

#### 2.2.1 Mechanisms of viral integration

Viral integration into the host genome is not a random event. Research has shown that integration sites often cluster with fragile sites in the genome, which may have implications for the activation of proto-oncogenes. It is orchestrated through a repertoire of intricate mechanisms, including non-homologous end joining (NHEJ) and microhomology-mediated end joining (MMEJ). The precise loci of these integration events can significantly differ across instances, determining the severity of genetic disruption and subsequent activation of oncogenes. Delving into the specific mechanisms employed by different viruses helps elucidate their unique contributions to cancer.

#### 2.2.2 Consequences of viral integration

Once viruses find their way into the human genome, their repercussions on cellular machinery are multifaceted: The integration of the viral genome into the host can be seen as a double-edged sword, with both benefits and detriments for both parties. Activation of Cellular Oncogenes: Viral DNA, when integrated, can strategically position viral enhancers or promoters in proximity to cellular proto-oncogenes. This can lead to the aberrant activation of genes like MYC in the case of HPV-associated cervical cancers, accelerating the journey to tumorigenesis. Disruption of Regulatory Elements and Pathways: Beyond the activation of proto-oncogenes, viral integration can also disrupt host genes, alter regulatory elements, or introduce novel oncogenes. Such disruptions can skew gene expression, compromise cellular function, and enhance cellular growth or survival tendencies. Inactivation of Tumor Suppressor Genes: Notably, viruses also have the ability to incapacitate tumor suppressor genes, such as TP53 and PTEN. The loss of these sentinels of cellular regulation paves the way for uncontrolled growth and genomic instability.

#### 2.2.3 Viral-induced genomic instability and clonal evolution

The integration of viral DNA into the host genome can lead to genomic instability, fostering an environment ripe for mutations. Certain viruses can directly induce DNA damage through their replication processes, leading to genomic instability. Viruses may interfere with host DNA repair mechanisms, compromising genome stability. This interference can result in the accumulation of DNA mutations, which are critical for clonal evolution. As these mutations accumulate, as the immune system exerts selective pressure on viral variants, certain cell clones with conferred growth advantages may begin to dominate the tumor landscape. Over time, the tumor evolves, becoming a heterogeneous entity teeming with sub-clones, each potentially harboring distinct viral integration patterns. In the context of antiviral therapy, viral-induced genomic instability can contribute to the emergence of drug-resistant viral strains. Understanding the dynamics of clonal evolution is essential for designing effective therapeutic strategies. Developing strategies to interfere with viral integration or eliminate integrated viral genomes could be a promising approach to reduce viral-induced genomic instability and its associated pathologies.

## 3 Third-generation sequencing technologies and viral integration

The genomic landscape of cancer has been revolutionized by advancements in sequencing technologies (13, 49). A significant facet of this change has been brought about by third-generation sequencing technologies, which illuminate the dynamics of viral integration into the human genome (50–52). Long-read sequencing allowed the sequencing of chimeric reads, incorporating both viral DNA and the host genome fragments on either side (53). This aids in detecting multiple clonal integration events and uncovering key features of viral integration in the cancer genome. The promise of these technologies lies in their capability to offer extended read lengths, real-time sequencing, and unprecedented resolution, all of which are pivotal in studying the intricate relationships between viral oncogenes, viruses, and cancer (54–57).

### 3.1 Pacific biosciences sequencing

PacBio sequencing has emerged as a pioneering technology in genomics, particularly for its unique capabilities in generating long reads. This groundbreaking approach enables researchers to delve deeper into the complexities of genomes, transcriptomes, and epigenomes, unlocking a wealth of biological information that was previously challenging to access. One of the standout features of PacBio sequencing is the ability to produce exceptionally long reads, often spanning thousands to tens of thousands of base pairs. This stands in stark contrast to short-read sequencing technologies, allowing for the detection of structural variants, complex genomic rearrangements, and complete characterization of repetitive regions that were previously elusive. Such extended read lengths are especially advantageous in the context of viral-related tumors, as they facilitate the precise mapping of viral integration sites within the human genome, offering a deeper understanding of the molecular events underpinning tumorigenesis. Moreover, PacBio sequencing excels in deciphering the epigenetic landscape through the analysis of DNA modifications, offering insights into the role of DNA methylation and histone modifications in viral-related tumors (58, 59). These capabilities make PacBio sequencing an invaluable tool for unraveling the intricacies of viral oncogenesis and hold significant promise for the development of advanced diagnostic and therapeutic strategies in the field of cancer research.

### 3.2 Nanopore sequencing

Another formidable player in the third-generation sequencing realm is nanopore sequencing, which involves the passage of DNA or RNA molecules through nanometer-sized pores, enabling real-time monitoring of nucleotide sequences (60–63). Its advantages include long-read capabilities, portability, and the potential for direct RNA sequencing. Beyond the capability to provide long reads, nanopore sequencing is distinctive due to its real-time, single-molecule sequencing of DNA strands. For studies central to viral integration, this technology holds promise by allowing direct observation of the integration process. Such insights grant researchers the capability to identify structural variants that have arisen due to viral integration, providing a more comprehensive understanding of the process. Nanopore sequencing has been instrumental in characterizing the integration patterns of the HPV genome into host DNA, providing insights into the molecular events driving cervical cancer (52, 64–66). Importantly, the study showed that within the same sample, separate integration events often clustered closely, with partial overlap at different breakpoints (52, 65).

### 3.3 Implications on epigenetics and functional genomics

Third-generation sequencing technologies, when juxtaposed with functional genomics methodologies like RNA-seq and chromatin immunoprecipitation sequencing (ChIP-seq), forge a powerful toolset. In the context of investigating CpG methylation in novel and existing transposable element (TE) insertions, both Oxford Nanopore Technologies and PacBio long-read sequencing techniques offer valuable capabilities (60, 67, 68). These sequencing platforms enable the analysis of CpG methylation patterns within TE insertions in a comprehensive and detailed manner. Specifically, researchers can employ ONT and PacBio long-read sequencing to capture long sequences and comprehensively cover TE insertions, many of which can be extensive and complex. Moreover, these long sequencing technologies can resolve methylation patterns and allow for precise mapping of CpG methylation, providing important insights into the epigenetic alterations associated with tumorigenesis and other biological processes (69, 70). This combination enables researchers to probe deeper, investigating the ramifications of viral integration on host gene expression, epigenetic shifts, and chromatin configurations. It is this intersection of technologies that holds the potential to unravel the mechanisms steering viral oncogenesis. Moreover, as sequencing technologies evolve, so do the methods for virus detection. Current and emerging molecular and immunological methods are expanding the toolkit for researchers, allowing for more comprehensive and accurate identification of viruses in various samples.

### 3.4 Analysis of HPV integration

Recent progress in sequencing technology has led to the capability of producing exceptionally long reads, averaging around 100 kb. This advancement includes the generation of high-throughput, full-length mRNA or cDNA reads and the construction of genomic contigs exceeding 100 Mb. However, this technological leap presents a challenge: existing alignment programs are either incapable or inefficient in handling such extensive data, highlighting the urgent need for new, more capable alignment algorithms. The field of bioinformatics has been evolving rapidly to meet these demands, with significant strides made in the development of alignment methods for long sequencing reads. These methods are particularly adept at chimeric sequence-aware alignment, crucial for identifying reads that align with both human and viral reference genomes – a key process in understanding pathogen-host interactions and disease mechanisms.

One standout tool in this domain is Minimap2 (71), a versatile alignment program designed to map DNA or long mRNA sequences against extensive reference databases. Alongside Minimap2, other notable aligners have emerged, including desalt (72), which is tailored for aligning long DNA or mRNA sequences. Additionally, tools originally developed for short reads are being adapted and enhanced for the unique challenges posed by error-prone long reads. This includes TAGET (73) and a newly adapted mode of the STAR aligner (73). These improvements reflect the ongoing evolution of bioinformatics tools, ensuring they remain at the forefront of managing and interpreting the burgeoning data from next-generation sequencing technologies.

## 4 Discussion

Viral-induced genomic instability and clonal evolution represent an intricate and multifaceted aspect of viral infections. Understanding the mechanisms and consequences of this phenomenon is essential for the development of effective therapeutic strategies. As our knowledge continues to expand, further research is needed to unveil new insights into this complex relationship and its potential implications for human health. Ultimately, deciphering the interplay between viruses and host genomes will be critical in advancing our ability to prevent and treat viral-associated diseases.

Third-generation sequencing technologies have ushered in a new era in the study of viral-related tumors, offering unprecedented insights into the molecular intricacies of viral oncogenesis. The intricate interplay between viruses, oncogenes, and human cancers remains a vital research frontier, with oncogenic viruses playing an undeniable role in global cancer epidemiology. Our understanding of this dynamic has been immensely enriched by third-generation sequencing technologies, which have cast light on the hitherto elusive mechanisms underlying viral integration into the human genome.

The advent of third generation sequencing has revolutionized our capacity to pinpoint viral integration sites with exceptional resolution, enabling a deeper comprehension of their implications on functional genomics. As the genomic landscape of cancer continually evolves, these technologies provide a dynamic lens, capturing shifts in viral integration sites over time and documenting tumor progression and heterogeneity. The ability to map viral integration sites, study epigenetic modifications, and track clonal evolution provides a multifaceted view of these complex diseases. The practical implications are vast, from innovative diagnostic markers to personalized treatment strategies and the exploration of novel therapeutic targets.

However, the significance of these insights extends beyond the domain of academic research. The translational potential of understanding viral oncogenesis is vast. Recognizing unique viral integration patterns can provide innovative diagnostic markers, heralding early detection and improved patient outcomes. The meticulous mapping of the relationship between viral integration and cellular response can pave the way for novel therapeutic strategies. The insights gained from third-generation sequencing in viral-related tumors hold significant potential for clinical applications. Here are some practical implications: 1) Innovative Diagnostic Markers: Third-generation sequencing can identify unique patterns of viral integration and epigenetic alterations in viral-related tumors. These patterns can serve as innovative diagnostic markers, allowing for the early detection and stratification of cancer subtypes. 2) Personalized Treatment Strategies: Understanding the clonal evolution and heterogeneity of viral-related tumors through third-generation sequencing helps in tailoring personalized treatment approaches. This can lead to more effective therapies and improved patient outcomes. 3) Exploring Novel Therapeutic Targets: The precise characterization of viral oncogenes and their interactions with the host genome can uncover novel therapeutic targets. Inhibiting viral-specific factors can be a promising avenue for targeted cancer therapies.4) Monitoring Treatment Response: Third-generation sequencing can be used to monitor the response of viral-related tumors to treatments over time. This real-time monitoring can guide adjustments in therapeutic strategies and help predict the course of the disease.

As we move forward, addressing the challenges of data analysis, integration with other omics data, validation in clinical settings, and cost considerations will be essential (74, 75). It is also important to integrate these findings with other functional and clinical data. This holistic approach will be essential in realizing the full potential of these insights, from bench to bedside. While third-generation sequencing has brought about remarkable advancements in the study of viral-related tumors, several challenges and future directions warrant attention. 1) Data Analysis and Interpretation: Managing the large volumes of data generated by third-generation sequencing is a considerable challenge. The development of robust bioinformatics tools and analytical pipelines is crucial to extract meaningful insights. 2) Integration with Other Omics Data: A holistic understanding of viral-related tumors often requires the integration of genomic, transcriptomic, proteomic, and epigenomic data. Future research should focus on methods for seamless integration of these multidimensional datasets. 3) Validation in Clinical Settings: To translate the findings from third-generation sequencing into clinical practice, rigorous validation studies are essential. These studies should assess the diagnostic and prognostic utility of the identified markers and therapeutic targets. 4) Cost and Accessibility: While third-generation sequencing technologies have advanced, their cost and accessibility remain barriers to widespread adoption. Efforts to reduce costs and improve accessibility will be instrumental in realizing the full potential of these technologies in clinical settings.

## Author contributions

RY: Conceptualization, Investigation, Writing – original draft, Writing – review & editing. AW: Data curation, Writing – original draft, Writing – review & editing. BB: Investigation, Writing – original draft, Writing – review & editing. PL: Data curation, Writing – review & editing. WD: Conceptualization, Data curation, Formal analysis, Investigation, Methodology, Resources, Software, Supervision, Writing – original draft, Writing – review & editing. XZ: Funding acquisition, Investigation, Methodology, Resources, Supervision, Writing – original draft, Writing – review & editing. SY: Conceptualization, Data curation, Formal analysis, Funding acquisition, Investigation, Resources, Supervision, Writing – original draft, Writing – review & editing, Methodology, Project administration, Software, Validation, Visualization.
